# Induced Pluripotent Stem Cell (iPSC)–Derived Lymphocytes for Adoptive Cell Immunotherapy: Recent Advances and Challenges

**DOI:** 10.1007/s11899-019-00528-6

**Published:** 2019-06-26

**Authors:** Alexandros Nianias, Maria Themeli

**Affiliations:** Department of Hematology, Amsterdam University Medical Centers, Cancer Center Amsterdam, Location VUmc, Amsterdam, Netherlands

**Keywords:** Induced pluripotent stem cells, Adoptive cell immunotherapy, NK cells, T cells, Off-the-shelf

## Abstract

**Purpose of Review:**

In the rapidly developing field of adoptive cell immunotherapy, there is urgent need for discoveries that would improve outcomes, extend the applicability, and reduce the costs. Induced pluripotent stem cells (iPSC) can be a source of broadly applicable cellular immunotherapeutics, which have been manufactured, validated, and banked in advance, and can be applied across HLA barriers. Here, we discuss the recent advances and challenges in the generation of iPSC-derived cellular products for cancer therapy.

**Recent Findings:**

iPSCs can be differentiated to functional tumor-specific T and NK cells in vitro with demonstrable in vitro and in vivo anti-tumor activity. Genetic modifications employed at the iPSC level can deliver desirable immunotherapeutic attributes to the generated immune effectors. iPSC-NK cells are currently evaluated in a clinical setting and pre-clinical testing of iPSC-T cells shows promising results but their production seems more challenging.

**Summary:**

The use of iPSCs for the generation of tumor-targeting T/NK cells constitutes a feasible strategy to overcome limitations in manufacturing, efficacy, and applicability of cellular therapeutics.

## Introduction

Adoptive cell immunotherapy for the treatment of hematological malignancies has greatly advanced in the last decades, leading to significant clinical outcomes. Both T cells and NK cells have been proven robust therapeutic agents and several approaches to obtain anti-tumor therapeutic lymphocytes have been proposed with variable clinical impact. These include the isolation and expansion of patient-derived tumor antigen-specific T cells (T cell clones, tumor infiltrating lymphocytes), innate T cells (NK-T or γδ-T cells), or NK cells [1, 2]. The introduction of genetic engineering with T cell receptors (TCR) and most importantly chimeric antigen receptors (CAR) boosted the applicability and efficacy of adoptive cell immunotherapy [3]. CARs are artificial receptors that redirect antigen recognition from T cells and mediate T cell activation, through the fusion of an extracellular antigen-binding moiety, such as a single-chain-variable region (scFv), with an intracellular signaling domain [4]. CAR-modified T cells (CAR-T) targeting CD19 have induced impressive responses in chemotherapy-resistant B cell leukemias and lymphomas [5, 6], while B cell maturation antigen (BCMA)–targeting CAR-T cells show promising clinical results against multiple myeloma [7]. These remarkable results led further to the development of CAR-engineered NK cells (CAR-NK), redirecting the intrinsic capacity of NK cells for tumor recognition and elimination and the initiation of CAR-NK clinical trials [8, 9].

Although successful, current approaches of adoptive cell therapy have significant limitations that impede their further progress and broader use. Immunotherapy using primary T cells is mainly performed in an autologous setting limiting a facile and general use. The production of genetically engineered therapeutic cells is a time-consuming process and the required processing time can be detrimental for the patient’s health. Also, in many cases, the autologous T cell isolation and expansion could be problematic and their functionality and quality doubtful (e.g., patients receiving highly immunosuppressive therapy). In addition, the existing ex vivo T cell expansion protocols push T cells to a terminal differentiated effector state, resulting in exhausted, less effective cellular products. The use of allogeneic cells from volunteer donors has the potential to broaden the applicability of the T cell products to HLA-matched recipients [10]. The advances of gene editing technology allow for further broadening of the applicability of CAR-T cell therapy by the use of allogeneic volunteer donor T cells that have been modified to lack TCR expression and thus, avoid graft-versus-host disease (GvHD) [10]. However, the final purity of the TCR-less CAR-T cell product is not always acceptable. Carriage of even < 1% (< 4–5 × 10^4^ cells/kg) of residual TCR-expressing T cells was still enough to initiate GvHD in the first clinical application of TCR-less CAR-T cells [10]. In addition, the potential off-target genetic alterations require further safety testing of every different batch produced that brings higher production costs. Extra gene editing to also eliminate HLA class I expression and reduce the risk of rejection could further reduce production efficiency (60% of double targeting efficiency) [10, 11] and require extra ex vivo expansion in order to reach the desirable yield of fully edited cells. However, it is known that longer ex vivo culture can be at the cost of having a more exhausted final product [12]. Notably, NK cells exert their function regardless of the recipient’s HLA haplotype and thus they can be isolated from an unrelated donor or cord blood. Clinical trials using adoptively transferred allogeneic NK cells showed limited toxic side effects such as GvHD [13–17]. However, additionally to the above-mentioned limitations, their inadequate proliferative capacity impedes their in vivo persistence without cytokine support [18] and makes multiplex gene editing challenging. Immortalized NK cell lines, such as NK92, have been used as an alternative but they have to be irradiated before being infused to the patient [9, 19]. This limits their in vivo survival and thus, multiple doses of high numbers of NK92 cells are required [9]. Clinical application of irradiated NK92 cells transduced with a CD33-CAR was safe but without impressive anti-tumor results [9].

The development of broadly applicable cellular therapeutics, which have been manufactured, functionally validated and banked in advance, and can be applied across HLA barriers would improve the consistency and availability and reduce the cost of adoptive cell therapy. The generation of human lymphocytes from iPSCs has attracted lately the interest of the scientific community since it offers tantalizing prospects for cell-based therapies serving as an endless supply of custom-made, “off-the-shelf” therapeutic lymphocytes. Here, we review the latest advances in generating therapeutic anti-tumor T and NK lymphocytes from human induced pluripotent stem cells (iPSCs) and future challenges towards the final development of universally applicable immunotherapeutic products.

## iPSCs as an “off-the-shelf” Supply of Therapeutic Lymphocytes

The iPSC technology offers new perspectives for the production of immunotherapeutic cellular products due to two major characteristics. First, similar to embryonic stem cells, iPSCs can be cultured unlimitedly in vitro and be successfully differentiated towards the lymphoid lineage [20]. Having access to constant and continuous production of T and/or NK lymphocytes offers solution to cell number and doses limitations due to restricted availability or expansion of primary cells. Second, iPSCs can be easily amenable to genetic transformations in vitro and thus, can generate immune effectors, which may eventually be genetically modified to augment their applicability, potency, and persistence. While the potential for multiplex gene editing is limited in primary cells, iPSC can theoretically bear unlimited genetic changes. Finally, in contrast to primary cells [10], genetic engineering of iPSCs results in fully modified clonal lines, which could be extensively evaluated resulting in a stable and safe source. The generation of safe master iPSC lines, bearing genetic modifications that confer the desired characteristics to the final product, would facilitate the development of “off-the-shelf” cellular therapeutics for more patients and types of malignancy.

**Fig. 1:**
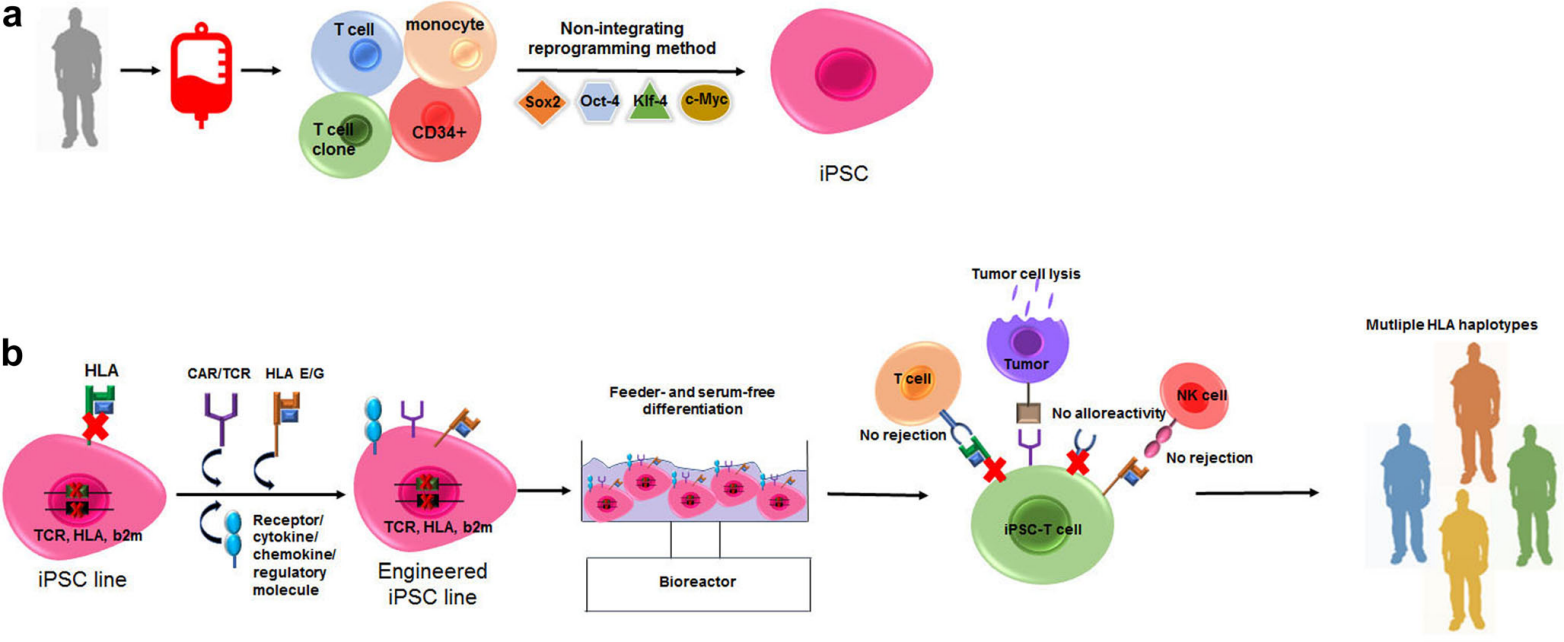
**a** Peripheral blood cells serve as a primary source to generate iPSC lines by non-integrating delivery of reprogramming transcription factors. **b** Generation of iPSC-derived off-the-shelf tumor-specific T cells. iPSCs are genetically modified to bear desirable immunotherapeutic properties. The expression of TCR and HLA is knocked out or silenced to prevent alloreactivity and graft rejection respectively. HLA-E/G molecules can be overexpressed to avoid NK cell–mediated transplant rejection, whereas antigen-specific TCR/CARs can direct anti-tumor activity. Further, introduction of the expression of immune receptors, cytokines, chemokines, or other immune regulatory factors may enhance anti-tumor function. Genome-edited master iPSC lines are differentiated under GMP-grade conditions to fully functional histocompatible tumor-targeting T cells accessible to all patients regardless of their HLA haplotype

Up to date almost all somatic cell types have been successfully reprogrammed into iPSCs through introduction of defined transcription factors (Oct4, Sox2, Klf4, c-Myc or Oct4, Sox2, Nanog, Lin28) [21••, 22]. However, the selection of the initial iPSC source seems to be important when aiming in the efficient generation of therapeutically relevant T or NK lymphocytes (Fig. 1a). Although, iPSC, from any somatic cell, can be successfully differentiated towards the lymphoid lineage, it has been shown that starting from blood cell-derived iPSCs, such as CD34^+^ cells from cord blood, monocytes or peripheral blood lymphocytes, results in a more efficient generation of CD4^+^CD8^+^ double-positive (DP) thymocytes in vitro [23••] suggesting a level of epigenetic memory [24]. Moreover, iPSCs from peripheral blood T lymphocytes (T-iPSC) have the unique characteristic of bearing the rearranged TCR loci of the parental cells, which remain unchanged during in vitro differentiation [21••, 23••]. Therefore, T cells with defined TCR-specificity (e.g., T cell clones, invariant T cells, NK-T cells) can be selected for reprogramming to T-iPSC for therapeutic T cell production.

## Generation of Customized, Anti-tumor T Lymphocytes from iPSCs

### Antigen Specificity and Other Characteristics

The hallmark of adoptive T cell immunotherapy is the use of the ability of T cells to specifically recognize tumor antigens. Naturally, this ability is endowed through the expression of specific TCRs encoded by the uniquely rearranged genomic loci of the TCRα and β chains. Generation of T cells from embryonic stem cells or iPSC, which bear non-rearranged germline TCR loci, results in random rearrangements during differentiation and a population of cells with various unknown specificities [25]. Nishimura et al. demonstrated that differentiation of T-iPSC–derived from an antigen-specific T cell clone gives rise to T cells with the same TCR rearrangement and reactivity [21••], thus making T-iPSC from tumor antigen-specific T cells a way to deliver defined specificity to iPSC-derived T cells. Until now, T cell clones specific for several tumor antigens, such as MART-1 (melanoma), LMP2 (EBV antigen), WT-1 (leukemia), and GPC3 (hepatocellular, ovarian, and lung carcinoma), have been reprogrammed to T-iPSC [23••, 26••, 27••]. Importantly, tumor-specific T-iPSC could give rise to cytotoxic CD8 single-positive (SP) T cells, which could recognize the target antigen on cell lines and display specific cytotoxicity [23••, 26••, 27••]. Although most of the generated cells bear the original tumor-specific TCR, it has been observed that TCRα can be additionally rearranged, resulting in TCR destabilization and loss of the antigen specificity [26••]. Inactivation of recombination activating gene 2 (*RAG2*) in the T-iPSC, a key protein complex in the rearrangement of TCR, can inhibit the process of further TCRα rearrangement at the DP stage and result in preservation of antigen specificity in CD8 T cells [26••].

Although taking advantage of a naturally occurring TCR in order to convey antigen specificity to iPSC-derived T cells, one can imagine that the flexibility of this method is limited, as the existence of pre-made, HLA-matched antigen-specific T cell clones is a requirement. The advances in engineering of immune cells open up new perspectives for the generation of custom-made synthetic T cells from iPSC. Antigen specificity can be assigned to iPSCs and their T cell derivatives by means of a transgenic TCR [26••]. Themeli et al. demonstrated the feasibility of generating functional CAR-T cells by engineering T-iPSC with a CAR [28••]. The use of CARs endows T-iPSC–derived T cells with HLA-independent, customizable antigen recognition as scFv domains of different specificity can be used giving the potential for a wider applicability range. Importantly, second- and third-generation CARs provide additionally costimulatory signals and enhance T cell activation, expansion, and in vivo persistence [4]. In principal, similarly to conventional CAR-T cells, T-iPSC can be also further genetically armed with cytokines, receptors, and other regulatory molecules in order to provide their derivatives with optimal immunotherapeutic properties such as enhanced proliferation and reduced exhaustion [29–32]. Especially the rise of gene editing technologies, such as CRISPR/Cas9 and TALEN technologies, offers new perspectives for the multiplex modification of T-iPSC.

### Differentiation Methods

The potential of iPSCs to become a valuable source of readily available anti-tumor T cells depends on the development of a defined and efficient production process that could yield the cell numbers required for clinical application. In 2009, Timmermans et al. first reported the derivation of mature CD3^+^TCR^+^ T cells from human embryonic stem cells [25]. Since then, several groups have demonstrated successful in vitro generation of T lymphocytes from iPSC, although using slightly different methods [21••, 23••, 28••]. However, all described protocols follow the same differentiation path, recapitulating the process of human T cell development. First, iPSCs are induced to form mesoderm from which definitive hemogenic endothelium arises as a next step. Hemogenic endothelium is then transitioning to form a pool of hematopoietic stem and progenitor cells (HSPC), a subpopulation of which has the potential to commit to the T cell lineage. The final important steps involve the emergence of CD8αβ^+^/CD4^+^ DP cells and eventually of mature CD8 or CD4 SP T lymphocytes. Since this is a multi-step differentiation process where every developmental transition happens with different efficiency, one could imagine that the production of mature SP T cells for clinical applications is a challenge. In recent studies, investigators were able to generate enough numbers of anti-tumor T cells in order to test their functionality in vivo in xenograft murine models [26••, 27••, 28]. However, the development of an efficient method for the production of clinically relevant cell numbers is still not reported.

Another major challenge is that, although successful in generating T cells from iPSC or T-iPSC, most of the reported differentiation methods include the presence of uncharacterized serum and feeder cells of murine origin, which are not compatible with clinical level production. Induction of the hematopoietic program has been achieved through co-culture with OP9, a murine bone marrow stromal cell line, or C3H10T1/2, a mouse embryonic fibroblast line [21••, 23••, 33]. Further T lymphoid commitment requires the use of the same cell lines of murine origin transduced to overexpress the Notch ligands DLL1 or DLL4 [20, 21••, 23••, 33]. One could replace the murine feeder cells with cells of human origin, but until now attempts to create human-origin feeder cells for T cell development had disappointing results, as human fibroblasts or keratinocytes have failed to efficiently support the differentiation of human CD34^+^ to pro T cells or SP mature cells [34, 35]. Therefore, the development of a feeder-free and serum-free method is required. Kennedy et al. managed to replace feeder use, for the differentiation of iPSC towards CD34^+^CD43^−^ HSPC, with a serum-free and stroma cell-free protocol based on embryoid body formation and the use of rationally selected cytokine combinations in a stepwise manner [20]. Creating an in vitro thymic niche by using plate-bound recombinant molecules of DLL4/DLL1 and VCAM-1, fused to the Fc portion of human IgG, has been used to generate T cell lineage cells from or cord blood-derived CD34^+^ cells [36], but the successful feeder-free differentiation of iPSC-derived HSPC has not been described.

### Phenotype and Functionality of iPSC-Derived T Cells

Beyond antigen specificity, the functional potential of T cells depends also on their developmental maturity and their lineage subtype (γδ, αβ, ΝΚ-Τ, CD4 or CD8, Treg, etc). This underscores once more the importance of developing differentiation protocols, which are based on the knowledge of human T lymphoid development, for the generation of the T cell subtype with the desired functionality. The first studies generating cytotoxic T cells from T-iPSC revealed that their phenotype and functionality was not precisely similar to that of mature CD8 T cells. The T-iPSC–derived rejuvenated T cells although they were CD3^+^TCR^+^ showed T cell–specific gene expression profile and elicited specific cytotoxic responses against cells expressing the target antigen; they lacked expression of important surface molecules (such as CD2, CD5, CD28) and expressed high levels of innate T cell-related markers (such as CD56) [21••, 23••]. Also, Themeli et al. further reported that CAR-modified T-iPSC differentiate into CD3^+^TCR^+^CD8αα^+^ CAR-T cells whose gene expression profile was similar to that of γδ-T cells [28••]. Importantly, their in vivo anti-tumor functionality was analogous to that of peripheral blood-derived γδ-T cells from the same donor bearing the same CAR [28••]. Therefore, although the generated T-iPSC–derived T cells express the endogenous αβ TCR, they have phenotypic and functional characteristics of an innate-like lymphocyte. Similar lineage skewing has been observed in transgenic TCRαβ mice [37–39] and in vitro differentiation of TCR-engineered human CD34^+^ hematopoietic progenitors [40], wherein the emerging TCRαβ^+^ T cells displayed innate T cell features, such as expression of CD8αα and low levels of CD5 [37]. CD8 is expressed as a CD8αβ heterodimer on mature cytotoxic T cells while CD8αα homodimers are present only on innate lymphocyte subtypes such as NK cells, γδ-T cells, NK-T cells, or intestinal epithelial lymphocytes (IEL) [41, 42]. Heterodimeric CD8αβ has been shown to be a better coreceptor for TCR/pMHC binding than homodimeric CD8αα [43] and the presence of CD8β is indicative of maturity. Indeed, T-iPSC–derived T cells expressing CD8αβ exhibited improved antigen-specific cytotoxicity in vitro and in vivo compared with CD8αα cells which show innate-like non-antigen-specific reactivity [27••].

It has been suggested that premature expression of the transgenic TCRαβ may prevent β-selection of the cells, skew development towards the γδ lineage, and result in the emergence of TCRαβ-expressing T cells with γδ properties [37, 38]. Interestingly, the pre-rearranged endogenous TCRαβ of T-iPSC is already expressed on day 15–20 of differentiation on OP9-DL1 [28••], which is remarkably earlier than the appearance of TCRαβ/γδ in differentiation of cord blood (CB)-CD34^+^ cells and some other reports on human ES/iPSC T cell differentiation [20, 25]. In addition, similarly to what is reported for the TCRαβ transgenic mice, the generation of CD8αβ-expressing DP cells from T-iPSC is very inefficient [21••, 27••, 33]. Interestingly, previous studies in transgenic murine models have demonstrated that lineage determination during T lymphoid differentiation is dependent on the synergy between TCR and Notch signaling and differences in Notch signal strength are also an important factor influencing αβ versus γδ development [44]. Murine T-iPSC differentiated in a 3D thymic culture generated antigen-specific anti-tumor T cells, which were phenotypically and functionally more similar to naïve CD8αβ T cells in contrast to CD8αα cells emerging from the OP9-DL1 co-culture [45•]. Therefore, a thymic environment provides the correct combination of Notch and other signals that promote the maturation of thymocytes. Further research should focus on the development of in vitro differentiation systems that better mimic the interactions and the balanced TCR and Notch signaling that takes place within the thymus.

Phenotypic and functional evaluation of the T-iPSC–derived CD8αβ T cells [27••] or iPSC-derived CD8αβ T cells bearing a transgenic TCR [26••] showed that they are similar to their peripheral blood counterparts. However, there are still gene expression discrepancies such as the lack of chemokine receptors (CCR7, CXCR3) and a weaker but still existing NK-like cytotoxicity [27••]. In addition, it is still not clear whether their anti-tumor function is equivalent to that of conventional CD8αβ T cells. The in vivo anti-tumor functionality of regenerated CD8αβ T cells has been up to date tested in xenograft models where the tumor cells are inoculated intraperitoneally or subcutaneously and thus, not in a naturally occurring location limiting our insight on their migratory capacities [26••, 27••, 28••, 33]. Finally, although treatment with iPSC-derived CD8αβ T cells significantly delayed tumor growth, it required multiple injections of high numbers of cells [26••, 27••, 33]. The use of xenograft models where tumor cells are inoculated at their “natural” sites and T cell-dose escalation could be more informative on the functionality of the iPSC-derived T cells.

## Generation of Therapeutic NK Cells from iPSCs

NK cells constitute a robust part of the innate immune system implicated in recognition and lysis of malignant and virally infected cells. Their HLA-independent cytotoxic capacity makes them favorable candidates for off-the-shelf cellular therapeutic product compared with T cells. However, as already mentioned, their limited proliferative and genetic manipulation potential render their clinical application challenging. The perspective of manufacturing therapeutic NK cells from iPSC provides solutions to many of the bottlenecks of adoptive NK cell therapy.

Interestingly, the generation of NK cells from iPSC has proven easier and more straight-forward than the production of antigen-specific cytotoxic T cells. Although the first steps of differentiation towards HSPCs are similar to that of T differentiation, the commitment to NK lymphoid lineage is less complicated and does not require the presence of Notch signaling. Several studies have demonstrated the robust production of homogeneous, mature NK cells from human iPSC, which express all significant NK-defining markers such as CD56, FcγRIIIa receptor (CD16), CD94, killer immunoglobulin-like receptors (KIRs), natural cytotoxicity receptors (NKp30, NKp44, and NKp46), activating receptors (NKG2D and DNAM-1), and death-inducing ligands (FasL, TRAIL) [46, 47•, 48••]. Most importantly, Knorr et al. reported a clinical-grade, serum-free, and feeder-free differentiation protocol to obtain functional NK cells from iPSC, which involves embryoid body formation in defined conditions and the use of membrane-bound interleukin 21-expressing artificial antigen-presenting cells [49]. According to the authors of the study, enough cytotoxic NK cells to treat a single patient could be produced from fewer than 250,000 input hiPSCs, thus facilitating the potential for clinical application in cancer therapy.

Apart from a mature phenotype, iPSC-derived NK cells display efficient cytotoxic capacity through direct receptor-mediated lysis, cytokine and chemokine secretion, and antibody-dependent cell-mediated cytotoxicity (ADCC) [46, 47•]. When evaluating the anti-tumor activity in an ovarian cancer xenograft model, intraperitoneally injected iPSC-derived NK cells showed similar delay of tumor progression and overall survival as peripheral blood NK cells expanded on artificial antigen-presenting cells (aAPC) [47•]. However, although statistically significant, this anti-tumor effect was not impressive and required multiple doses of NK cells. Interestingly, engineering iPSCs with a CAR bearing NK-specific costimulatory domains derived from NKG2D and 2B4 proteins optimized the targeted anti-tumor activity of the generated CARiPSC-NK cells and improved their in vivo expansion and cytotoxic capacity [48••]. Importantly, a single dose of CARiPSC-NK cells resulted in less toxicity but similar anti-tumor effect as third-generation CAR-T cells against ovarian cancer in vivo, although comparison of tumor burden was limited to 3 weeks post infusion in this study [48••]. Further genetic engineering has been proposed in order to improve the function and therapeutic potential of iPSC-NK cells. For example, the expression of a non-cleavable CD16 would enhance ADCC potential, the addition of an IL15Rα-IL-15 fusion can provide self-stimulation, and expression of CXCR3 can improve homing of iPSC-NK cells [50].

## Universal Off-the-Shelf Therapeutic Lymphocytes

Although generation of T and NK cells from iPSC overcomes many of the limitations of current manufacturing practices, their use would only really facilitate the applicability of adoptive cell therapy if they are available as a true universally applicable “off-the-shelf” product, which could be infused to any patient.

The major barrier limiting the applicability of allogeneic iPSC-derived products is the HLA-disparity between the effector T cells and the host, which may lead to graft rejection or graft-versus-host (GvH) reaction. Using matched previously banked iPSCs from HLA-homozygous donors as a starting material has been previously proposed [51]. It has been calculated that an iPSC bank with 50 HLA-homozygous iPSC lines could cover approximately 73% of the Japanese population [51] while 93% of the UK population would find a match within 150 HLA-homozygous lines [52]. However, the establishment of “universal” iPSC lines is considered to provide a “true” solution as they would provide widely applicable cellular products without the need for HLA-matching. Hypoimmunogenic, histocompatible pluripotent stem cell lines can be generated by elimination of HLA class I and II expression by disruption of β2m and CIITA gene respectively [53••, 54••, 55••]. Allogeneic cells, which lack “self” class I HLA molecules, can however be rejected by host NK cells. Introduction of HLA-E, HLA-G, or of patient-specific HLA-C has been shown to reduce NK-mediated rejection of iPSC-derived cells [53••, 54••, 55••]. Introduction of additional immunomodulatory molecules such as PD-L1 and CD47 can further reduce the recipients’ immune responses [55••].

When using allogeneic T cells, the possibility of graft-versus-host reactions is a major concern. In order to avoid alloreactivity of iPSC-derived T cells, T-iPSC bearing an endogenous TCR of known specificity (virus- or cancer-specific) could be used. Alternatively, the surface expression of the TCR could be disrupted by the means of genome editing as previously described for conventional CAR-T cells [10, 56].

Although, many of the above genetic modifications have been already reported and evaluated in the iPSC level, there is up to date no study showing the generation of functional and mature lymphocytes from universal iPSCs.

## Conclusions

The advancement of adoptive cell immunotherapy and the impressive clinical outcomes obtained targeting hematologic malignancies with CAR-T cells dictate for further developments towards a broader use of cellular therapeutics for more patients and more types of malignancy. The advent of iPSC technology provides new perspectives for the manufacturing of customized, tumor-targeting T/NK cells, with improved immunotherapeutic properties and the potential of universal “off-the-shelf” use (Fig. 1b). Rapid progress in the field of lymphoid differentiation of iPSC has brought the clinical application of iPSC-derived adoptive immunotherapy from theory to reality. Indeed, the first clinical trial testing an off-the-shelf, iPSC-derived NK cell product against advanced solid tumors started recruiting in 2019 (ClinicalTrials.gov Identifier: NCT03841110). In addition, the production of iPSC-derived T cells and TCR/CAR-engineered T cells is already in pre-clinical development. Fate Therapeutics is developing TCR-less T-iPSC–derived CD19-CAR-T cells where the CD19CAR is expressed from the TCR α chain constant region (TRAC) locus, while Adaptimmune aims to develop off-the-shelf anti-tumor T cells from TCR-engineered iPSC.

However, there are still several challenges to be pre-clinically addressed before the first clinical application of iPSC-derived T cells. As mentioned above, the phenotypic and functional maturity of the generated T cell effectors has to be ensured as well as an anti-tumor potential comparable with natural T cells. Further, manufacturing protocols should be established which would allow for the efficient, GMP-grade, and clinical scale production of iPSC-derived T cell products. Finally, as with all iPSC-derived cellular products, the potential risk of malignant transformation due to contamination with undifferentiated iPSC has to be minimized, for example with the use of suicide genes such as the iC9/CID system [33]. Further future advances in iPSC and genome editing technologies in combination with in-depth knowledge of the fundamental mechanisms of T/NK cell function and the regulation of lymphoid development will provide the tools for the generation of iPSC-derived T/NK cell products with improved therapeutic anti-tumor function, better homing, persistence, and applicable across histocompatibility barriers.
